# A ventricular fibrillation cardiac arrest model with extracorporeal cardiopulmonary resuscitation in rats: 8 minutes arrest time leads to increased myocardial damage but does not increase neuronal damage compared to 6 minutes

**DOI:** 10.3389/fvets.2023.1276588

**Published:** 2023-11-03

**Authors:** Alexandra-Maria Stommel, Sandra Högler, Matthias Mueller, Ingrid Anna Maria Magnet, Petra Kodajova, Benjamin Ullram, Alexander Szinovatz, Felix Paul Panzer, Anna Engenhart-Seyrl, Julia Kaschmekat, Tamara Schütz, Michael Holzer, Wolfgang Weihs

**Affiliations:** ^1^Department of Emergency Medicine, Medical University of Vienna, Vienna, Austria; ^2^Department of Pathobiology, Unit of Laboratory Animal Pathology, University of Veterinary Medicine Vienna, Vienna, Austria; ^3^Center for Biomedical Research and Translational Surgery, Medical University of Vienna, Vienna, Austria

**Keywords:** ventricular fibrillation cardiac arrest, rat model, extracorporeal cardiopulmonary resuscitation, neuronal damage, myocardial damage, global cerebral ischemia

## Abstract

**Introduction:**

Extracorporeal cardiopulmonary resuscitation (ECPR) is an emerging strategy in highly selected patients with refractory cardiac arrest (CA). Animal models can help to identify new therapeutic strategies to improve neurological outcome and cardiac function after global ischemia in CA. Aim of the study was to establish a reproducible ECPR rat model of ventricular fibrillation CA (VFCA) that leads to consistent neuronal damage with acceptable long-term survival rates, which can be used for future research.

**Materials and methods:**

Male Sprague Dawley rats were resuscitated with ECPR from 6 min (*n* = 15) and 8 min (*n* = 16) VFCA. Animals surviving for 14 days after return of spontaneous resuscitation (ROSC) were compared with sham operated animals (*n* = 10); neurological outcome was assessed daily until day 14. In the hippocampal cornu ammonis 1 region viable neurons were counted. Microglia and astrocyte reaction was assessed by Iba1 and GFAP immunohistochemistry, and collagen fibers in the myocardium were detected in Azan staining. QuPath was applied for quantification.

**Results:**

Of the 15 rats included in the 6 min CA group, all achieved ROSC (100%) and 10 (67%) survived to 14 days; in the 8 min CA group, 15 (94%) achieved ROSC and 5 (31%) reached the endpoint. All sham animals (*n* = 10) survived 2 weeks. The quantity of viable neurons was significantly decreased, while the area displaying Iba1 and GFAP positive pixels was significantly increased in the hippocampus across both groups that experienced CA. Interestingly, there was no difference between the two CA groups regarding these changes. The myocardium in the 8 min CA group exhibited significantly more collagen fibers compared to the sham animals, without differences between 6- and 8-min CA groups. However, this significant increase was not observed in the 6 min CA group.

**Conclusion:**

Our findings indicate a uniform occurrence of neuronal damage in the hippocampus across both CA groups. However, there was a decrease in survival following an 8-min CA. Consequently, a 6-min duration of CA resulted in predictable neurological damage without significant cardiac damage and ensured adequate survival rates up to 14 days. This appears to offer a reliable model for investigating neuroprotective therapies.

## 1. Introduction

Sudden out-of-hospital cardiac arrest (OHCA) is a major health burden with poor outcome (1). In Europe, the incidence rate of OHCA in which cardiopulmonary resuscitation (CPR) was attempted fluctuates between 21 and 91 cases per 100,000 population annually (2).

Animal models have been instrumental in discovering novel therapeutic strategies to mitigate injuries caused by global ischemia and reperfusion following extended, untreated cardiac arrest (CA) (3, 4). In recent years, the Vienna resuscitation research group has focused on developing a rodent model of ventricular fibrillation cardiac arrest (VFCA) (5, 6).

Extracorporeal cardiopulmonary resuscitation (ECPR) has gained prominence as a rescue therapy for patients in CA, refractory to conventional measures of CPR (7). While resuscitation research often emphasizes traditional CPR techniques, especially in VFCA rodent models, small animal VFCA models employing ECPR are seldom mentioned in existing literature. Consequently, we developed a VFCA ECPR rat model (8). Briefly, rats with VFCA underwent ECPR following 10 minutes (min) of CA, with targeted temperature management at 33°C for 12 hours (h) post-return of spontaneous circulation (ROSC). Our model demonstrated high survival rates and favorable neurological outcomes in the ECPR group as compared to the conventional CPR group, although we have acknowledged the challenges in implementing such an intricate resuscitation technique in a rat model (9).

The objective of this study was to create a reliable and replicable ECPR VFCA rat model capable of exhibiting acceptable long-term survival rates while still reflecting profound neuronal damage. We sought to pinpoint the precise duration of VFCA needed to attain this, comparing 6 and 8 min of VFCA and 14 days (d) of survival as study endpoint. Beyond resuscitation with ECPR, no additional therapeutic interventions were employed, and animals were maintained at normothermic conditions during ECPR and after ROSC. This model is anticipated to be an appropriate platform for future research on the pathophysiological mechanisms underlying ischemic damage and the exploration of corresponding therapeutic strategies. Our primary outcome focused on histological brain damage in the cornu ammonis 1 (CA1) region of the hippocampus, while secondary outcomes included survival rate, neurologic outcome, and myocardial damage resulting from global ischemia and reperfusion after a 14-day survival period.

## 2. Materials and methods

### 2.1. Animals and experimental protocol

The experimental protocol complied with the Austrian Federal Law (§ 16 Tierversuchsgesetz, TVG 2012), Directive 2010/63/EU and the ARRIVE guidelines (10), and was approved by the Institutional Ethics Committee for Laboratory Animal Experiments of the Medical University of Vienna and the Federal Ministry of Education, Science and Research of Austria (GZ: BMBWF-66.009/0315-V/3b/2018).

**Figure 1:**
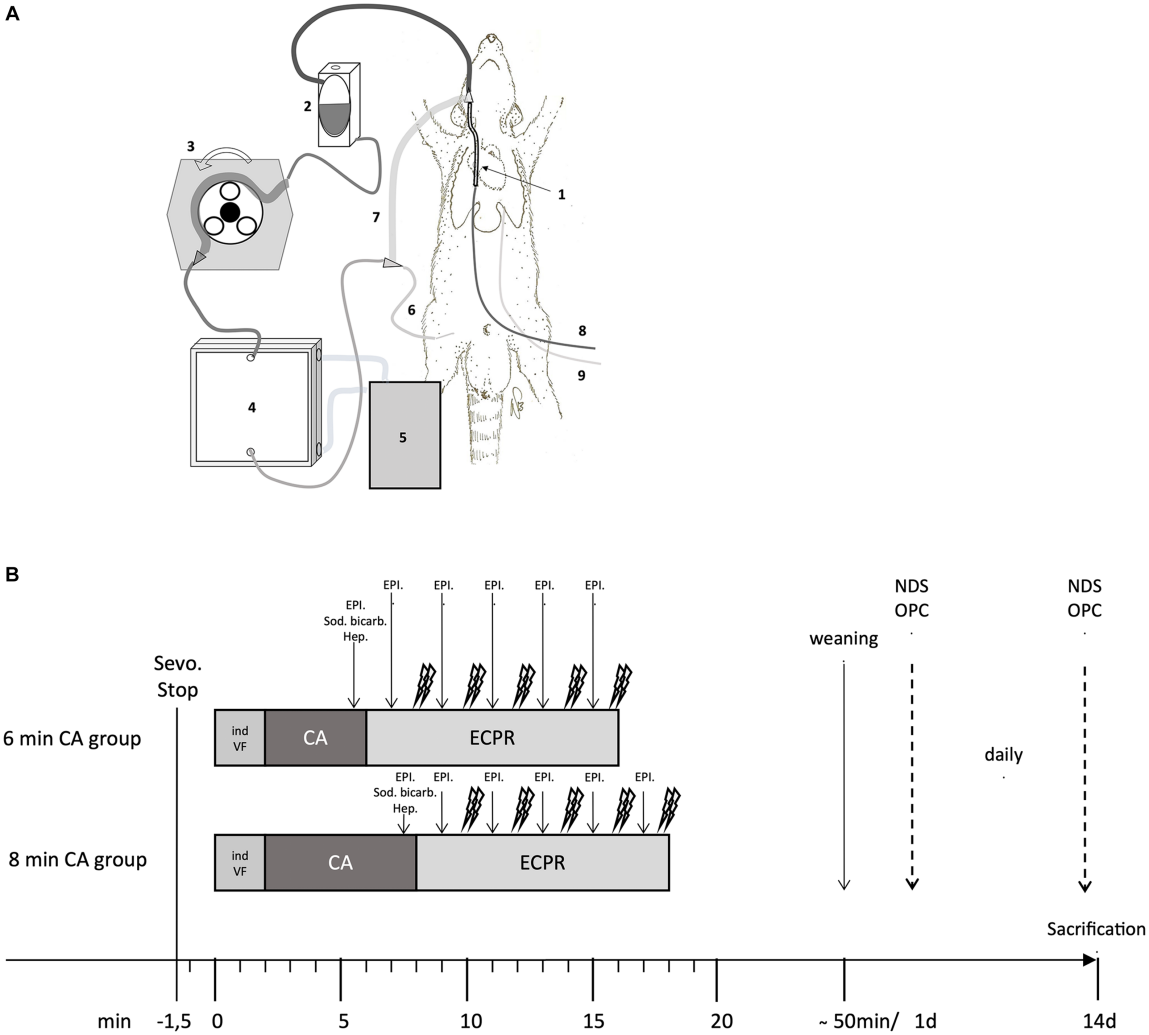
**(A,B)** ECMO set-up in the rat and Timeline of the experimental protocol. **(A)** Extracorporeal membrane oxygenation (ECMO) circuit consisting of a venous ECMO drainage cannula (1) inserted in the right jugular vein, an open reservoir (2), a roller pump (3), an oxygenator (4) with attached heat exchanger (5) and an arterial ECMO cannula (6) inserted in the right femoral artery. All these elements (custom-made; Dipl.-Ing. Martin Humbs, Valley Germany) were connected by a tubing system with shortcut (7) for priming before resuscitation. Medication was given via left femoral venous cannula (8), invasive blood pressure was measured and blood gas samples collected via left femoral arterial cannula (9). **(B)** Timeline of the experimental protocol in the 6 min and 8 min cardiac arrest (CA) group; Sevoflurane stop (Sevo. Stop) 1.5 min bevor inducing of ventricular fibrillation (ind VF); Followed by cardiac arrest (CA) for 6 or 8 min; Extracorporeal cardiopulmonary resuscitation (ECPR); heparin (Hep.); epinephrine (EPI.); sodium bicarbonate (Sod. bicarb.); daily scoring of Neurologic Damage Score (NDS) and Overall Performance Category (OPC); sacrification at day 14.

Male Sprague–Dawley rats, aged 10–12 weeks with a bodyweight (BW) of 450–600 grams, were obtained from the Division of Laboratory Animal Science and Genetics, Himberg, Austria. The animals were acclimated at the Center of Biomedical Research of the Medical University of Vienna for at least 14 d prior to the study. Rats were subjected to VFCA and resuscitated using ECPR. After a 14-day survival period, the animals were euthanized, and their brain and heart tissues were examined histologically and immunohistochemically. Details of the ECPR rat model used can be found in previous publications (8, 11), and the extracorporeal membrane oxygenation (ECMO) set-up is depicted in Figure 1A and Supplementary Figure S1.

### 2.2. Anesthesia and preparation

Rats were anesthetized using 6% inhaled sevoflurane (FiO_2_ 1.0) and received piritramide (3 mg/kg BW, s.c.) for analgesia. They were endotracheally intubated (adapted venous cannula, 14GA Venflon BD Luer-Lok, Helsingborg, Sweden) and anesthesia continued with 3.5% sevoflurane. Mechanical ventilation (Harvard Inspira advanced safety ventilator, Holliston U; volume controlled; RR 65/min, 7 mL/kg BW, 0.3 FiO_2_) was maintained, and the animals were continuously monitored for physiological parameters (3-lead ECG, esophageal and rectal temperature probe, invasive arterial blood pressure, etCO_2_, SpO_2_). They were placed on a heated operating table to maintain a body temperature of 37 ± 0.2°C during the experiment.

In the femoral vessels the arterial ECMO cannula (custom-made cannula, 22-gauge; inserted 2 cm in the right femoral artery), the venous and arterial catheter for venous drug application, hemodynamic monitoring and arterial blood gas sampling were implanted (Argyle 2.5-french umbilical neonatal vessel catheter; inserted 9 cm in the left femoral vein and 11 cm in the artery), using aseptic techniques. The venous ECMO drainage cannula (custom-made; 5 cm length; Dipl.-Ing. Martin Humbs, Valley, Germany) was inserted via the right jugular vein with the tip in the inferior vena cava (correct placement verified by sonography). All catheters and cannulas were heparinized and after insertion of the venous ECMO cannula a heparin bolus (500 IU/kg BW) was administered intravenously (i.v.) to avoid clotting. The ECMO set-up (custom-made; Dipl.-Ing. Martin Humbs, Valley, Germany) was primed (crystalloid solution, 15 mL Elo-Mel isotone Infusionslösung, Fresenius Kabi, Bad Homburg, Germany) and connected to the arterial ECMO cannula (Figure 1A; Supplementary Figures S1, S2). In the venous ECMO drainage cannula a pacing catheter (neonatal pacing catheter; Vygon GmbH & Co Bi-Pacing-ball 3 Fr, Aachen, Germany) was inserted to induce VFCA. Defibrillation paddles (Heartstart Pediatric Plus Pads, Philips, Andover, Mass) were size-adapted and stitched to the left and right side of the thorax. Baseline arterial blood gas analysis was conducted.

### 2.3. Cardiac arrest and resuscitation

Sevoflurane administration was halted 90 s prior to the end of ventilation and simultaneous initiation of VF (alternating electrical current of 12 V/50 Hz at a max. of 8 mA applied via the pacing catheter lying in the right heart) as previously described by Janata et al. (12) VFCA was defined as VF pattern in the ECG curve and drop of systolic aortic pressure < 20 mmHg with corresponding loss of arterial pulsation in the arterial pressure monitoring. This definition stays in accordance with the Utstein Style Guidelines for Animal Resuscitation (13). After removal of the pacing catheter the ECMO tubing was connected to the venous drainage cannula and priming medication (Heparin 200 I.U./kg BW; epinephrine 20 μg/kg BW; sodium bicarbonate 1 mmoL/kg BW) was added to the open reservoir of the ECMO. After either 6 or 8 min of untreated CA, ventilation and ECPR were initiated (100% oxygen, gas flow 100 mL/min; ECMO flow rate started with 30 mL/min/kg BW and increased to 100 mL/min/kg BW). After 2 min of ECPR, defibrillation with 2 biphasic shocks à 5 Joule (Heart-Start MRx Defibrillator; Philips, Andover, Mass) were administered every 2 min of resuscitation if shockable rhythm was present; mechanical ventilation (RR 20/min; FiO_2_ 1.0) was continued and epinephrine (10 μg/kg BW) was administered i.v. every 2 min during ECPR until ROSC was achieved or resuscitation efforts were halted after a maximum of 10 min of ECPR. The protocol is outlined in Figure 1B.

### 2.4. ROSC, explantation phase and intensive care phase

Upon achieving ROSC (defined as organized cardiac rhythm and the return of pulsations in the arterial pressure curve) (8), the ECMO was halted, and the blood from the ECMO circuit was returned via the arterial in-flow cannula. Ventilation was immediately (RR 65/min; FiO_2_ 0.5) adapted. Prompt removal of the venous ECMO cannula occurred after ROSC, and the wound was closed aseptically. “Sustained ROSC” was defined as maintenance of mean arterial pressure (MAP) exceeding 60 mmHg for a duration of 20 min without mechanical support. Arterial blood gas analyzes were conducted at intervals of 5 min, 15 min and 60 min following ROSC, with ventilation adapted as required. Throughout this period, continuous monitoring of the rats was performed as they remained on the heated operating table until the femoral catheters were removed. The rats were then successfully weaned from the ventilator and extubated.

During the initial 24–48 h post-resuscitation, the rats were predominantly unconscious and therefore housed individually until recovery. Analgesia was provided with piritramide (3 mg/kg BW s.c.) at 6-h intervals as long as animals showed signs of pain. Supplemental NaCl 0.9% and Glucose 10% was given s.c. if the animals were unable to drink. Unconscious rats were placed on a heating pad with an integrated control unit linked to a rectal temperature probe to maintain normothermia.

For animals that had not fully recovered and required care, visits were scheduled at a minimum of 6-h intervals. These visits included administering pain medication, providing fluid therapy, and assisting with hand- or tube-feeding. Animals that were awake but too weak to eat unaided, were fed a nourishing blend of applesauce and finely ground cereals via gavage.

The sham group, which underwent all surgical interventions without experiencing CA and ECPR, received the same treatment duration and medications, excluding those specific to resuscitation.

Neurologic outcomes were assessed daily using the Overall Performance Category (OPC) (14) and the Neurologic Deficit Score (NDS) (15), respectively. The OPC was used to gauge neurological status (OPC 1 = normal, OPC 2 = moderate disability, OPC 3 = severe disability, OPC 4 = comatose, OPC 5 = dead) and is presented for all animals that achieved ROSC. The NDS (0–10% = normal; 100% = brain death) is presented for animals surviving until the study endpoint 14 d post arrest.

On the 14^th^ day, the animals were anesthetized and analgesized (with sevoflurane and piritramide), endotracheally intubated and mechanically ventilated. A thoracotomy was performed, and the heart was excised from the thorax and the brain was carefully removed from the skull. One randomly selected half of the brain, along with the whole heart, were preserved in a 4% buffered formaldehyde solution for histological analysis.

### 2.5. Histology and immunohistochemistry

Coronary brain sections containing the hippocampus were embedded in paraffin wax. Sections 2 μm thick were cut and stained with hematoxylin and eosin (HE) for descriptive evaluation and to count viable pyramidal neurons in two 250 μm long sectors of the hippocampal cornu ammonis 1 (CA1) region at 200x magnification.

Immunohistochemistry was performed to stain microglia and astrocytes using primary antibodies against ionized calcium-binding adapter molecule 1 (Iba1) and glial fibrillary acidic protein (GFAP), respectively. A polymer detection system, consisting of a secondary antibody conjugated to an enzyme-labeled polymer, was used in an autostainer (Lab Vision AS 360, Thermo Fisher Scientific, Waltham, United States). For details regarding pretreatment, antibodies, and dilution see Supplementary Table S1.

To assess microglia and astrocyte activation immunohistochemical stainings were evaluated in a descriptive manner and analyzed using QuPath, a software platform for bioimage analysis (QuPath-Quantitative Pathology & Bioimage Analysis, Version 4.2.3 for Windows, University of Edinburgh, United Kingdom). For this purpose, a digital image was taken of the hippocampal CA1-region by an Olympus SC50 camera mounted on an Olympus BX53 microscope with a 20x objective. In QuPath a pixel classifier was trained and applied to detect pixels stained brown in the immunohistochemical stainings (16).

For the histological assessment of the myocardium a coronary section of the heart at the major circumference was embedded in paraffin wax and cut at a thickness of 2 μm. These sections were stained with HE for descriptive evaluation and with Azan-staining following the Haidenhein method to identify collagen fibers. Assessment of myocardial scar formation was conducted by scanning whole slides (Panoramic SCAN II SC150-213005, 3DHISTECH KFT, Budapest, Hungary) and analyzing with QuPath to quantify the extent of collagen fibers. The myocardium was annotated, and a pixel classifier was trained and applied to the full slide scans to detect pixels stained blue in the annotated areas. The ratio of blue-stained pixels to all stained pixels was subsequently calculated.

### 2.6. Statistics and data analysis

For our statistical analysis, continuous data that were normally distributed are expressed as the mean ± standard deviation, while categorical variables are presented as counts and percentages. Ordinal data are described by the median and interquartile range.

For testing the null hypothesis of no difference between independent groups, we employed the Student’s *t*-test, Kruskal-Wallis-Test and Mann–Whitney-U-Test, as appropriate. Multiple comparisons were adjusted using Bonferroni corrections, and survival analysis was conducted using Kaplan–Meier methods.

For histological and immunohistochemical data, we first assessed normality using the Shapiro–Wilk Test. Normally distributed data were analyzed with ANOVA, while non-normally distributed data were analyzed using the Kruskal-Wallis-Test. Corrections for multiple comparisons were applied using Tukey’s range test and Dunn’s test, respectively.

Sample size calculation was performed for the primary outcome of histologic damage, CA1 hippocampus at 2 weeks survival. Assuming a slightly larger standard deviation of 7 one needs 10 animals per group do detect a difference of 10 cells with a power of 80% at a significance level of 0.05 (5). Dropout rates in earlier experimental groups have been 35% (6), therefore we included an additional number of 5 per group.

Data management and analysis were carried out using MS Excel, GraphPad Prism (software version 10.0.1 for Windows) and SPSS (version 28, SPSS, IBM Corporation, Somers, NY). We considered a two-sided *p*-value of less than 0.05 as indicative of statistical significance.

## 3. Results

### 3.1. Animal groupings and survival outcomes

**Figure 2:**
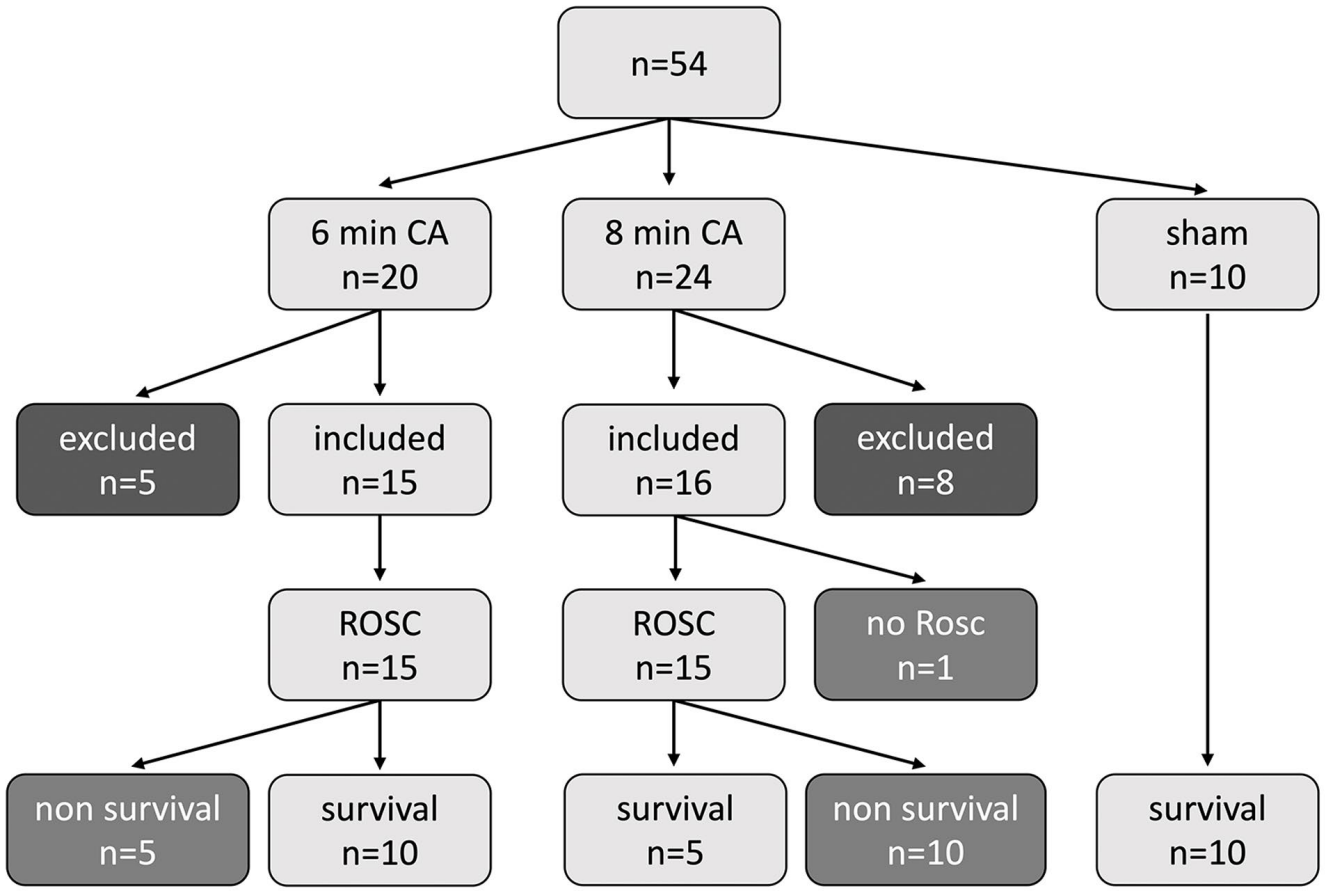
Study flowchart showing all animals (*n* = 54) used in the study. In the 6 min (min) cardiac arrest (CA) group of the 20 allocated rats, 5 animals were excluded for technical failure and 15 were included in the data analysis. All 15 rats achieved return of spontaneous circulation (ROSC), 10 rats survived until final study endpoint, 5 rats died before 14 d endpoint. In the 8 min CA group of the 24 allocated rats, 8 animals were excluded for technical failure and 16 were included in the data analysis. 15 rats achieved ROSC; 1 animal had no ROSC. 5 rats survived until final study endpoint, 10 rats died before. In the sham group 10 animals were assigned and survived until endpoint.

A total of 54 animals were distributed among two VFCA groups (6 min CA, *n* = 20; 8 min CA, *n* = 24), and one sham-operated group (sham, *n* = 10). Technical failure led to the exclusion of 13 animals, with 5 rats from the 6 min CA group and 8 rats in the 8 min CA group. In the 6 min CA group, all 15 rats included achieved ROSC (100%), and 10 (67%) survived to 14 days (453 ± 22 g at BL, 447 ± 29 g at sacrifice). Out of 16 animals included in the 8 min CA group, 15 (94%) achieved ROSC, with 5 (31%) surviving to 14 days (565 ± 46 g at BL; 534 ± 78 g at sacrifice; Figure 2).

**Figure 3:**
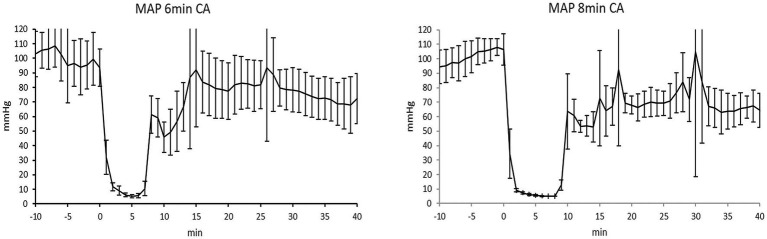
Mean arterial pressure in the 6 min CA group and 8 min CA group. Mean ± SD of mean arterial pressure (MAP), (mmHg) of the 6 min cardiac arrest (CA) group (MAP 6 min CA) and MAP of the 8 min CA group (MAP 8 min CA). Timeline shows MAP from 10 min (−10 min) before CA to 40 min after CA.

Hemodynamic data of the two arrest groups during CA and resuscitation are presented in Figure 3. Table 1 contains details on preparation and resuscitation phase. Blood gas parameters at the “Baseline,” 5 min, 15 min and 60 min post-ROSC are provided in Supplementary Tables S2–S5. Values of blood gas analyzes did not differ between the 6 min CA and 8 min CA groups but reflects the expected impact of CA and resuscitation in comparison to the sham group.

**Table 1:** Procedure and resuscitation details.

Procedure, Resuscitation	6 min CA	8 min CA	Sham
Time- SevoBox- (min)	6.3 ± 2.1	7.7 ± 3.5	8.1 ± 3.2
Time-preparation (min)	85.7 ± 20.7	76.2 ± 18.3	74.8 ± 19
mA max Fibrillation	5.2 ± 1.3	4.1 ± 1.4	0 ± 0
Defibrillation attempts	2 (IQR 2;4)	2 (IQR 2;4)	0 (IQR 0;0)
Epi-dosages	1 (IQR 1;3)	1 (IQR 1;3)	0 (IQR 0;0)
Resuscitation duration (min)	3 (IQR 2;5)	3 (IQR 3;5)	0 (IQR 0;0)
Survival (h)	312 (IQR 45;360)	77 (IQR 55;336)	336 (IQR 336;342)

6 min CA group, 8 min CA group, sham group: Anesthesia induction time (Mean ± SD), animals were placed in sevoflurane box for induction of sedation (Time- SevoBox- min); total preparation time (Mean ± SD), (min). Resuscitation: Induction of ventricular fibrillation cardiac arrest using maximum current (mA) (Mean ± SD). Median (IQR) of defibrillation attempts, shocks administered. Median (IQR) of epinephrine (Epi) dosages given during resuscitation phase. Median (IQR) of resuscitation duration in minutes (min). Median (IQR) of survival in hours (h) after Return of Spontaneous Circulation (ROSC).

**Figure 4:**
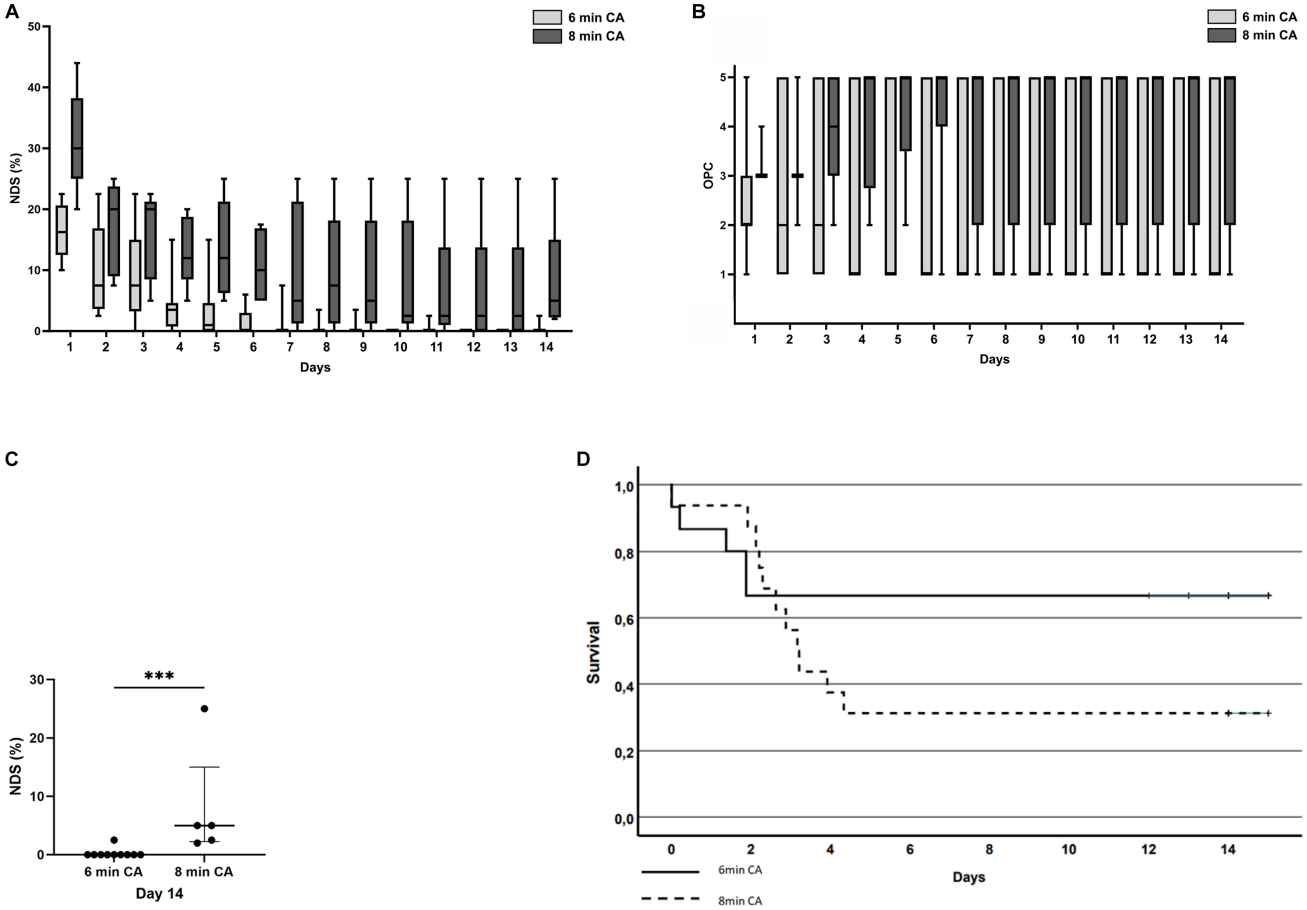
**(A)** Neurologic Damage Score (NDS, %) of all animals surviving until study endpoint; light gray bars = 6 min CA group, dark gray bars = 8 min CA group. **(B)** Overall Performance Category (OPC) score development over time of all animals that achieved ROSC; light gray bars = 6 min CA group, dark gray bars = 8 min CA group. **(C)** Neurologic Damage Score (NDS, %) of surviving animals at study endpoint (day 14); Significantly worse neurological outcome in 8 min CA group compared to 6 min CA group (*p* = 0.001), each dot represents an animal. **(D)** Kaplan–Meier analysis of cumulative survival at 14 days post resuscitation. Solid line = 6 min CA group; dashed line = 8 min CA group.

Neurologic outcomes were assessed daily using NDS and OPC scores, respectively (Figures 4A,B). On day 14 the neurological outcome of animals subjected to 8 min CA was significantly worse compared to 6 min CA animals in OPC (Table 2) and NDS scores (Figure 4C). The OPC score in the 6 min CA group was 1 [IQR 1;5], while in the 8 min CA group the OPC score was 5 [IQR 2;5] (*p* = 0.038). The NDS score was 0 [IQR 0;0] in the 6 min CA group compared to 5 [IQR 2.25;15] in the 8 min CA group (*p* = 0.001).

**Table 2:** Overall performance category (OPC) score at final examination and resuscitation success.

OPC score-final examination according to study groups	6 min CA	8 min CA	Sham
OPC 1	••••• •••••	•••	••••• •••••
OPC 2		•	
OPC 3		•	
OPC 4			
OPC 5	•••••	••••• •••••	
No ROSC		•	

OPC 1 = normal, OPC 5 = dead; no return of spontaneous circulation achieved (no ROSC). OPC score on final examination day, 14 days after cardiac arrest in the 6 min cardiac arrest group (6 min CA), 8 min cardiac arrest group (8 min CA) and sham group (sham). Each dot represents an animal.

The survival rates at 14 days did not differ significantly between the 6 min and 8 min CA group (*p* = 0.227). Cumulative survival analysis is depicted in Kaplan–Meier curves in Figure 4D.

### 3.2. Histological analysis and immunohistochemistry

#### 3.2.1. Hippocampus

**Figure 5:**
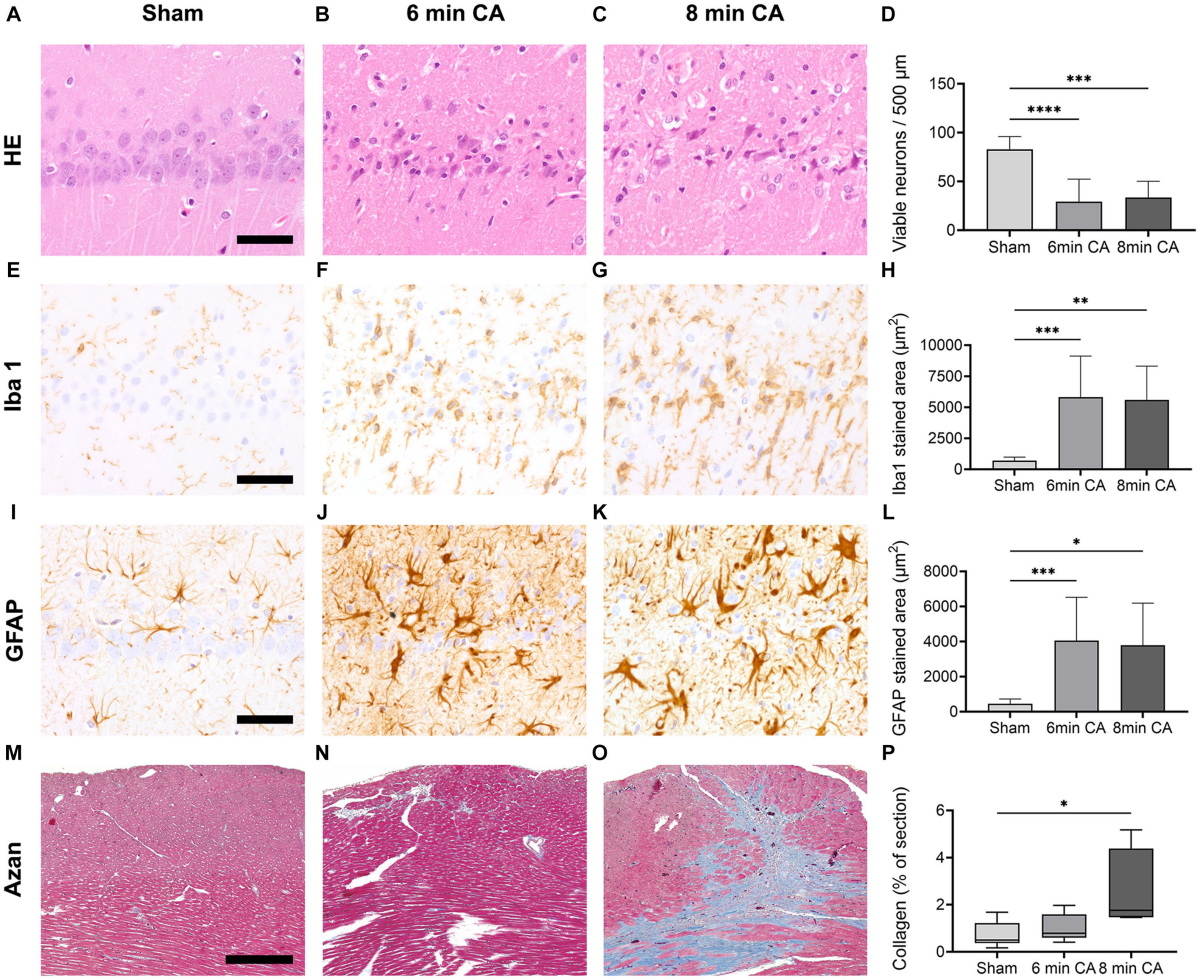
Representative images of histological and immunohistochemical stainings and according graphs of the quantitative evaluation, **** *p* < 0.0001, *** *p* < 0.001, ** *p* < 0.01, * *p* < 0.05. **(A–D)** HE-staining, bar = 50 μm: physiologic appearance of the hippocampal CA1 region with multiple viable neurons in a sham animal **(A)** compared to few viable neurons in a 6 min **(B)** and 8 min **(C)** CA animal, respectively; sham animals show significantly more viable neurons compared to CA animals [**(D)**, mean ± SD] **(E–H)** Iba1 immunohistochemistry, bar = 50 μm: resting microglia with small round nuclei and fine processes in a sham animal **(E)** compared to highly reactive microglia with rod shaped nuclei, increased cytoplasm, and few plump processes in a 6 min **(F)** and 8 min **(G)** CA animal, respectively; the stained area is significantly increased in CA animals compared to sham animals [**(H)** mean ± SD] **(I–L)** GFAP immunohistochemistry, bar = 50 μm: physiologic appearance of astrocytes in the CA1 region with little cytoplasm and fine processes in a sham animal **(I)** compared to highly reactive astrocytes with increased cytoplasm and plump processes in a 6 min **(J)** and 8 min **(K)** CA animal, respectively; the stained area is significantly increased in CA animals compared to sham animals [**(L)** mean ± SD] **(M–P)** Azan-staining to detect collagen fibers, bar = 300 μm: viable myocardium (red) with very little, predominantely perivascular, autochtonous collagen (blue) in a sham animal **(M)** compared to a 6 min CA animal with a small focus of collagen in the left upper quadrant **(N)**, while the 8 min CA animal shows multiple large coalescing foci of collagen fibers **(O)**; collagen in the myocardium of 8 min CA animals was significantly increased compared to sham animals [**(P)** Median ± IQR].

In the hippocampus of all animals subjected to CA either multiple hypereosinophilic, necrotic neurons were present, or many neurons were missing and replaced by activated astrocytes and microglia in HE-staining (Figures 5B,C). In contrast, the hippocampus of sham animals showed regular appearance with viable neurons in multiple layers in the CA1 region (Figure 5A). CA-animals showed a significant loss of viable neurons in the CA1 region of the hippocampus compared to sham animals in HE-staining (Figure 5D). In sham animals 83 ± 13 viable neurons were detected in two 250 μm long sections of the hippocampal CA1 region, while in animals with 6 min CA 29 ± 23 (*p* < 0.001) intact neurons and in animals with 8 min CA 34 ± 17 (*p* < 0.001) viable neurons were detectable. No statistically significant difference was found between the two cardiac arrest groups (*p* = 0.90).

Iba1 immunohistochemistry in the hippocampus of sham animals showed normal appearance of resting microglia with long fine branching processes and round nuclei surrounded by very little cytoplasm. In both CA groups microglia in the hippocampus was highly activated, characterized by increased cytoplasm, short plump processes, and rod like nuclei (Figures 5E–G). The area of microglia stained by Iba1 immunohistochemistry was significantly increased in both CA groups compared to sham animals (sham: 697 ± 286 μm^2^; 6 min CA: 5805 ± 3316 μm^2^, *p* = 0.001; 8 min CA: 5595 ± 2722 μm^2^, *p* = 0.004), but no differences were detected between the CA groups (*p* = 0.99) (Figure 5H).

Astrocytes, stained by GFAP immunohistochemistry, showed moderate amounts of cytoplasm and long slender processes in the hippocampus of sham animals consistent with physiological morphology. In contrast, astrocytes in both CA groups showed plump short processes and an increase in area and staining intensity (Figures 5I–K). This finding was confirmed by quantitative evaluation of the stained area. Both CA groups showed significantly more GFAP-positive pixels compared to sham animals (sham: 451 ± 275 μm2, 6 min CA: 4048 ± 2475 μm^2^, *p* < 0.001, 8 min CA: 3785 ± 2402 μm^2^, *p* = 0.012). No statistically significant differences were detectable between both CA groups (*p* = 0.97) (Figure 5L).

#### 3.2.2. Myocardium

The myocardium of sham animals exhibited normal morphology, with striated myofibers, small amounts of interstitial connective tissue present in perivascular, subendocardial, and subepicardial regions in HE staining. In contrast, myocardium after 8 min of CA showed multifocal to coalescing areas of activated fibroblasts producing eosinophilic fibrillar material associated with myofiber loss. Similar lesions were present in small, scattered foci in the myocardium of 6 min CA animals. The quantification confirmed a significant increase of Azan-stained collagen fibers in the 8 min CA group (1.76% of myocardium [IQR 1.47;4.39]) compared to the sham group (0.50% IQR [0.37,1.22], *p* = 0.012). No differences were detected between both CA groups (*p* = 0.27) or between 6 min CA group with 0.79% of myocardium [IQR 0.60;1.59] and sham group (*p* = 0.34). Representative images of all groups are shown in Figures 5M–P.

## 4. Discussion

In the present study, we utilized a normothermic ECPR model to compare two different durations (6 min and 8 min) of VFCA. The aim was to develop a model that would simultaneously create profound neuronal injury and maintain acceptable mortality rates. This would be suitable for future investigations into the pathophysiological mechanisms of ischemic damage as well as the development of therapeutic strategies.

In our comparison, the 6 min CA group showed a two-thirds survival rate to the endpoint with a more favorable neurologic outcome, while two-thirds of the 8 min CA group died. There were no differences in hippocampal neuronal damage between the groups, but the longer ischemia led to increased myocardial damage.

ECPR in rodent models is seldom reported in the literature and is mainly applied in asphyxia (17–20) or exsanguination models (21, 22). Even though experimental normovolemic VFCA better simulates adult human patients’ CA situation (23), comparable ECPR rat models are scarce (8, 12). Thus, we established a rat ECPR-VFCA model, evaluating the effects of global ischemia and resuscitation after two different CA durations on ROSC, 14-day survival rates, neurological outcomes, and histological damage in both brain and myocardium.

The successful resuscitation rates in our model (100% of the 6 min CA group and 94% for the 8 min CA group) highlight the feasibility of ECPR in achieving ROSC in most animals. The 33% pre-endpoint mortality rate in the 6 min group aligns with average drop-out rates in similar 6 min VFCA rodent models (24, 25), whereas the 63% mortality in the 8 min group underscores the severity of an additional 2 min of CA.

Janata et al. (12) used 6 min VFCA with ECPR and had reported similar ROSC and survival rates in their normothermic ECPR groups. Notably, in our previous 10 min VFCA ECPR rat model (8) with 100% ROSC rate we treated the resuscitated animals with targeted temperature management (33°C) for 12 h leading to a 90% survival rate after 14 d. In a publication of our group, Warenits et al. (9) described and discussed the difficulties and special challenges of implementing a demanding 10 min VFCA ECPR model. In the current study, we avoided cooling the animals, intending to create an untreated control model for researching the underlying processes related to ischemia duration and to enable future therapy research. The 14-day survival endpoint was crucial to observe the development of maximal neuronal damage in the CA1 region. This is the time span necessary for the maximal neuronal damage in the CA1 region to develop, as shown by Yasuda et al. (26) in their 10 min occlusion rat model.

In our former study, Schober et al. compared ECPR and conventional CPR after 8 min of VFCA in their rat model. In the ECPR group 5 of 8 animals had sustained ROSC. However, this was a short-time survival experiment to analyze metabolic changes during ischemia and resuscitation displayed by cerebral microdialysis. Per protocol the survival time in this study was 80 min from ROSC (11).

The final NDS and OPC scores were significantly different between the 6 min group and 8 min group. In the 6 min CA group, animals died within the initial 48 h, but subsequently, all surviving animals recovered. Conversely, in the 8 min CA group animals died until day 5, thereby prolonging the unstable phase in this group. Throughout this critical period of pronounced impairment, the animals progressively lost motor skills, and an escalating paresis was observed in all four extremities. During this time, animals were provided with intensive care and regular pain management.

In our study, animals exposed to 6- and 8-min CA exhibited a significant reduction in the number of viable neurons in the hippocampal CA1 region, relative to sham animals. This findings mirrors the results reported by Janata et al., who observed a similar decrease in neuron numbers in their ECPR rat model after 6 min of CA and a 14-day survival period (12). In contrast, a heterogenic outcome was found in our previous ECPR rat model with 10 min CA followed by 12 h of mild hypothermia. In this model, some animals demonstrated extensive damage while others remained nearly unaffected (8). The hypothermia treatment may account for these variations, as lesions appeared more uniform in the current study. In an asphyxia CA ECPR model a reduction of neurons was confirmed after 8 min of CA and 3 d of survival in the hippocampus of moderately cooled rats, which is in accordance with our results (17).

Interestingly, after 14 d of survival neither neuron numbers nor activation of microglia or astrocytes were different in the CA groups. An explanation for this lack of difference might be that neuronal damage and glial response after 6 min of CA are already maximized and longer duration of ischemia does not lead to increased lesions. This theory is consistent with our prior CPR model, where differences in neuronal damage were not significant between 6 min CA and 8 min CA groups, though a broader variation in intact neurons was found in the hippocampal CA1 region in the 6 min CA group (6). A limitation to consider is that only 5 out of 16 animals survived to the 14-day endpoint after 8 min of CA, potentially excluding those with more severe lesions from histological examination.

Acute myocardial necrosis was not detected after 14 days of survival. The presence of activated fibroblasts and the significant increase in collagen fibers following 8 min of VFCA indicates scar formation, a likely result of prior myocardial infarction. The 6 min CA group, in contrast, did not display a significant increase in collagen fiber content within the myocardium when compared to sham animals. This additional 2-min duration of CA appears to inflict greater damage to the myocardium, possibly contributing to the lower survival rate in the 8 min CA group. Tsai et al. documented fibroblasts proliferation and collagen deposition in the left ventricular myocardium in a 5 min VFCA CPR model with 72 h survival (27). This damage may be attributable to the resuscitation method, as we found a significant increase in collagen deposits only in animals subjected to 8 min of CA, suggesting that our ECPR treatment might cause less harm.

Furthermore, we detected a statistically significant elevation in microglial activation in the hippocampal CA1 region in both CA groups compared to sham animals. This observation corresponds with the findings of Janata et al. (12) in their VFCA CPR/ECPR model after 14 days of survival and Yasuda et al. (26) in a 10 min occlusion model. We also noted astroglial activation after 6 and 8 min of CA, corroborated by our CPR rat model with 8 min of CA (5). While other VFCA ECPR models have not specifically reported astrocyte reactions (8, 17, 21), Sadelli et al. (28) also described astroglial scar formation in the hippocampal CA1 region using a four-vessel occlusion model. These results collectively suggest that CA consistently triggers an inflammatory response in the hippocampus, irrespective of the specific arrest model or resuscitation technique employed.

## 5. Limitations

Due to logistical factors, the mean weight was higher in the 8 min CA group than in the 6 min CA group. While this does represent a limitation, we do not believe that weight differences within this range significantly impact the results. The reduced survival in the 8 min group led to a smaller number of animals available for histological comparison.

Additionally, some of the myocardial damage observed in our model may have been induced by the cardiac arrest induction procedure using a fibrillation catheter. However, only minor damage was detected in the 6 min CA group, and no damage was found in the control (sham) animals, which were exposed to the fibrillation catheter without current. This indicates that the cardiac arrest procedure itself is unlikely to be the sole cause of the observed myocardial damage.

Our ECPR rat model presents certain limitations when juxtaposed with the clinical scenarios in humans. Clinically, ECPR is reserved for a selected group of CA patients unresponsive to standard CPR efforts. Conversely, our animal model implants the ECMO prior to inducing CA. The ECMO is then initiated immediately after a 6 or 8-min no-flow period, bypassing the simulation of conventional CPR and the consequent low-flow period. In real-world clinical settings, ECPR is only applied if standard CPR is ongoing.

In our study, rats achieved ROSC within few minutes of ECPR and were promptly weaned off ECMO, followed by an immediate removal of all ECMO cannulas. This contrasts sharply with clinical procedures where weaning and decannulation are not undertaken in such early phase. Typically, ECMO management in human ECPR patients spans 3–4 days during the post-resuscitation period (29).

Although histologic examination of the hippocampal CA1 region is a standard evaluation of neuronal damage in CA models, we cannot rule out that additional damage in other brain regions differed between 6- and 8-min CA, contributing to the survival rate and/or longitudinally assessed neurological scores.

## 6. Conclusion

In this study using a VFCA ECPR model with 6- and 8- min no-flow CA durations, we demonstrated that extending the CA duration did not increase histological damage to the hippocampus in animals that survived for 14 days. However, the longer CA duration did result in a reduced survival rate. Consequently, a 6-min CA duration led to consistent neurological damage while maintaining satisfactory survival rates up to 14 days, positioning it as a stable model appropriate for studying neuroprotective treatments.

## Data availability statement

The raw data supporting the conclusions of this article will be made available by the authors, without undue reservation.

## Ethics statement

The animal study was approved by Institutional Ethics Committee for Laboratory Animal Experiments of the Medical University of Vienna. The study was conducted in accordance with the local legislation and institutional requirements.

## Author contributions

A-MS: Writing – original draft, Writing – review & editing, Data curation, Investigation, Conceptualization, Methodology, Supervision. SH: Investigation, Methodology, Writing – original draft, Writing – review & editing, Data curation, Formal analysis, Supervision, Visualization. MM: Investigation, Writing – review & editing. IM: Investigation, Writing – review & editing, Data curation. PK: Investigation, Writing – review & editing. BU: Investigation, Writing – review & editing. AS: Writing – review & editing, Investigation. FP: Writing – review & editing, Investigation. AE-S: Writing – review & editing, Data curation. JK: Writing – review & editing, Investigation. TS: Supervision, Writing – review & editing, Resources. MH: Data curation, Visualization, Writing – review & editing, Formal analysis. WW: Conceptualization, Investigation, Project administration, Resources, Supervision, Writing – original draft, Writing – review & editing.
