# Supplementary material for: A ventricular fibrillation cardiac arrest model with extracorporeal cardiopulmonary resuscitation in rats: 8 minutes arrest time leads to increased myocardial damage but does not increase neuronal damage compared to 6 minutes
Source: Front Vet Sci. 2023 Nov 3;10:1276588. doi: 10.3389/fvets.2023.1276588 (PMC10655001; doi:10.3389/fvets.2023.1276588)
Supplement: Supplementary file 1 [file Data_Sheet_1.pdf]

Supplementary Material

1. Supplementary Figures

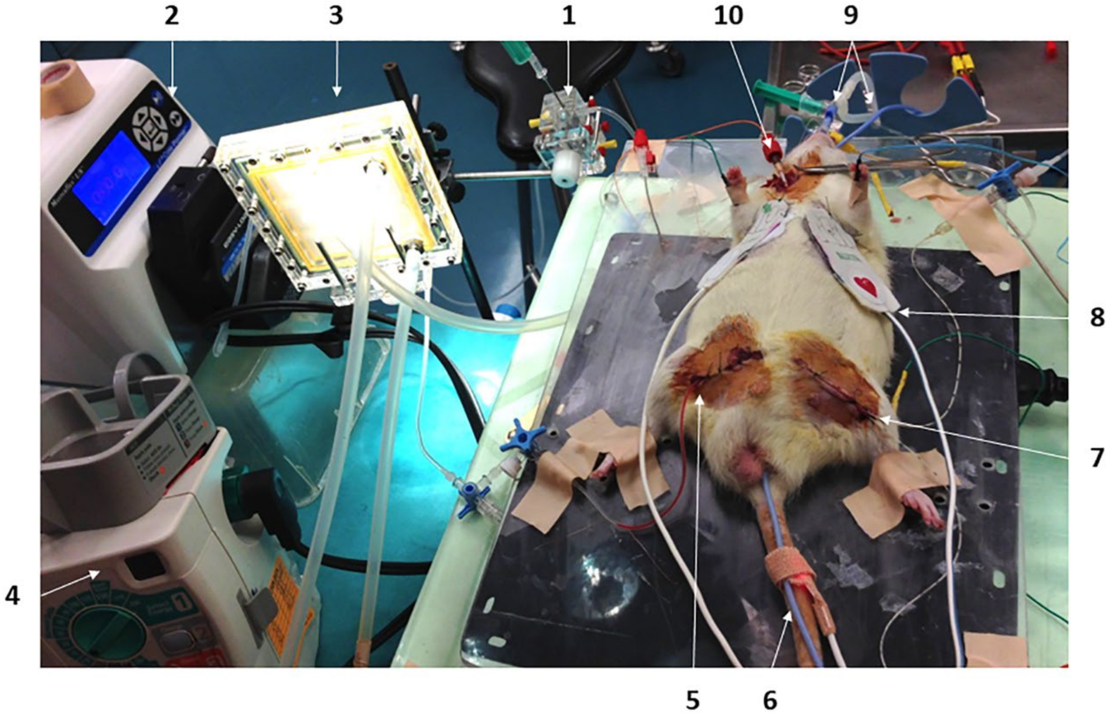

Supplementary Figure 1.

Extracorporeal cardiopulmonary resuscitation (ECPR) set-up in the rat before inducing cardiac arrest.

The anaesthetized, ventilated rat is lying on a heated table before inducing cardiac arrest. 1 open reservoir with inserted syringe and needle; 2 roller pump; 3 oxygenator connected with tubes to heat exchanger; 4 defibrillator; 5 arterial ECMO cannula access, wound provisionally closed during resuscitation; 6 rectal temperature probe; 7 arterial and venous access, wound provisionally closed during resuscitation; 8 defibrillator electrodes adapted in size; 9 endotracheal tube and esophageal temperature probe; 10 venous drainage cannula in the right jugular vein with inserted fibrillation wire, tube connection to the open reservoir not plugged in at the moment and set aside.

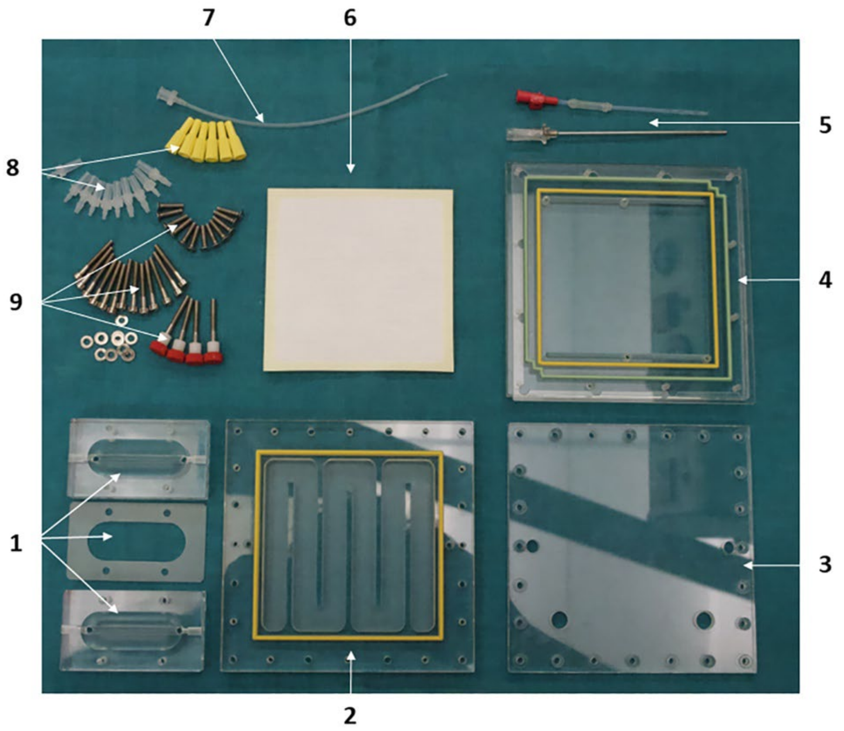

Supplementary Figure 2.

Parts of the ECMO oxygenator (Dipl.-Ing. Martin Humbs, Valley Germany) and arterial and venous ECMO cannula, assembled before each experiment; acrylic glass parts and metal parts cleaned and sterilized before reuse.

1 parts of open reservoir; 2 part of the oxygenator containing the milled pipes of the heat exchanger water; 3 outer cover of the heat exchanger lines; 4 casing part between which and part 2 the oxygenator membrane is inserted; 5 venous outflow cannula; 6 oxygenator membrane; 7 arterial ECMO cannula; 8 stoppers and connectors; 9 connecting screws and flat washers.

1. Supplementary Tables

Supplementary Table 1

Antibodies, dilution, antigen retrieval, and chromogen used for immunohistochemistry.

Immunohistochemistry				
**Primary Antibody**	**Antibody Dilution**	**Antigen Retrieval**	**Secondary Antibody**	**Chromogen**
**Iba 1**<br>Rabbit AB Wako (019-19741)	1:30.00<br>30 min RT	pH 6 citrate buffer<br>97°C 20 min	Bright Vision Goat anti Rabbit HRP RTU (KL DPVR 110 HRP)	DAB Quanto (Thermo Scientific #TA-125-QHDX)
**GFAP**<br>Rabbit AB Dako (Z0334)	1:5000<br>30 min RT	Pronase<br>37°C 5 min	Bright Vision Goat anti Rabbit HRP RTU (KL DPVR 110 HRP)	DAB Quanto (Thermo Scientific #TA-125-QHDX)

Supplementary Table 2

Baseline arterial blood gas parameters in the 6 min CA group, 8 min CA group and sham group (Mean ± SD). Parameters: pH (PH); pCO2 mmHg (PCO2); pO2 mmHg (PO2); Base Excess mMol/L (BE); hemoglobin g/dL (Hb); sO2 % (sO2); potassium mMol/l (K+); glucose mg/dL (Gluc); lactate mMol/L(Lac).

Base line blood gas values

	6 min CA	8 min CA	sham
PH	7.39±0.03	7.41±0.04	7.43±0.03
PCO2	35±5	33±6	33±5
PO2	168±21	170±36	180±44
BE	-3.1±2.8	-3.7±3.7	-1.8±2.7
Hb	11.9±1.4	11.1±1.7	11.9±1.4
sO2	96.2±3	96.2±1.7	96.5±1.4
K+	3.6±0.5	4.1±0.4	3.9±0.3
Gluc	148±41	135±36	157±58
Lac	1.2±0.3	1.3±0.4	1.2±0.2

Supplementary Table 3

Arterial blood gas analysis 5 min post ROSC in all study groups. Mean ± SD of the 5 min post Return of Spontaneous Circulation (pROSC) arterial blood gas parameters in the 6 min CA group, 8 min CA group and sham group. Parameters: pH (PH); pCO_2 mmHg (PCO2); pO_2 mmHg (PO2); Base Excess mMol/L (BE); Hemoglobin g/dL (Hb); sO_2 % (sO2); potassium mMol/l (K+); glucose mg/dL (Gluc); lactate mMol/L(Lac).

5 min pRosc			
	6 min CA	8 min CA	sham
PH	7.04±0.08	7.10±0.08	7.40±0.02
PCO2	44±6	44±8	38±2
PO2	184±72	165±51	141±62
BE	-17.1±3.9	-14.6±3.3	-1±1.5
Hb	8.6±1.2	9.1±1	12.6±0.3
sO2	92±6	101±44	96±1
K+	3.8±0.8	4±0.6	4.4±0.3
Gluc	260±74	258±57	133±23
Lac	9.2±2.2	8.6±1.6	1.2±0.6

Supplementary Table 4

Arterial blood gas analysis 15 min post ROSC in all study groups. Mean ± SD of the 15 minutes (min) post Return of Spontaneous Circulation (pROSC) arterial blood gas parameters in the 6 min CA group, 8 min CA group and sham group. Parameters: pH (PH); pCO_2 mmHg (PCO2); pO_2 mmHg (PO2); Base Excess mMol/L (BE); Hemoglobin g/dL (Hb); sO_2 % (sO2); potassium mMol/l (K+); glucose mg/dL (Gluc); lactate mMol/L (Lac).

15 min post Rosc			
	6 min CA	8 min CA	sham
PH	7.18±0.06	7.23±0.07	7.41±0.02
PCO2	37±5	36±5	37±3
PO2	206±66	195±36	145±30
BE	-13.3±3.3	-9.8±6.9	-1.1±2.4
Hb	10±1.3	11.2±1	12.4±0.7
sO2	96±2	96±2	97±2
K+	3.1±0.8	3.8±0.4	4.4±0.4
Gluc	218±34	273±57	137±32
Lac	6±1.9	7.5±2.4	1.1±0.6

Supplementary Table 5

Arterial blood gas analysis 60 min post ROSC in all study groups. Mean ± SD of the 60 minutes (min) post Return of Spontaneous Circulation (pROSC) arterial blood gas parameters in the 6 min CA group, 8 min CA group and sham group. Parameters: pH (PH); pCO_2 mmHg (PCO2); pO_2

mmHg (PO2); Base Excess mMol/L (BE); Hemoglobin g/dL (Hb); sO_2 % (sO2); potassium mMol/l (K+); glucose mg/dL (Gluc); lactate mMol/L (Lac).

60 min post Rosc			
	6 min CA	8 min CA	sham
PH	7.36±0.06	7.39±0.04	7.44±0.03
PCO2	33±4	38±8	34±7
PO2	189±57	184±74	164±29
BE	-6.2±4.7	-2.3±3.3	-1.3±3.3
Hb	9.9±0.8	11.2±1.3	11.8±1.6
sO2	96±2	94±5	97±1
K+	3.6±0.8	4.5±0.5	4.2±0.5
Gluc	130±41	138±49	150±30
Lac	2.2±1.6	2.9±2.8	1.1±0.4
